# Effect of an app for promoting advance care planning and motivating patients to write their advance directives

**DOI:** 10.1186/s12913-023-09593-3

**Published:** 2023-06-01

**Authors:** Céline Schöpfer, Catherine Bollondi, Mohamed Amir Moussa, Johanna Sommer, Christine Clavien

**Affiliations:** 1 Institute for Ethics, History, and the Humanities, University of Geneva, Geneva, Switzerland; 2 Direction of Care, University Hospitals of Geneva, Geneva, Switzerland; 3 Institute of Family and Child Medicine, Faculty of Medicine, University of Geneva, Geneva, Switzerland

**Keywords:** Mobile app, Advance directives, Advance care planning, ACP engagement, Patients

## Abstract

**Background:**

*Accordons-nous*, a smartphone app, was developed to support patients in the advance care planning (ACP) process. The app raises awareness and facilitates communication on this sensitive topic. It helps patients express their values and preferences for care and write their advance directives (AD).

**Objective:**

Measure the impact of distributing *Accordons-nous* on patients’ propensity to engage in the ACP process, compared with the distribution of a leaflet. A secondary objective was to test the effect of socio-demographic factors (age, health status, gender, level of education) on propensity to engage in ACP.

**Methods:**

Pre-post randomized control study. Participants were patients approached in medical waiting rooms. They received the app (treatment) or an information leaflet (control). They responded to two questionnaires: one at recruitment and a second 3–4 weeks later. Improvement on four variables relevant to ACP was measured: reported *contemplation* of an event relevant to ACP; *decision* about treatment in case of that event; *discussion* about it with relatives or health care professionals; *writing* advance directives. Statistical analysis included between-group comparisons of pre-post differences with 2-sample tests for equality of proportions and logistic regression models.

**Results:**

Four hundred seventy three participants were recruited and full responses obtained from 312. Overall, the intervention (control and treatment together) had a positive effect on the mean reported ACP engagement for all variables: new or renewed *contemplation* 54%; increase in *decision* 8%, *discussion* 11%, and *writing* 1%, compared to the baseline. Compared to the control (leaflet), the treatment group (app) had a larger effect size for all variables: between-group difference in *contemplation* + 11% (logistic regression, *p* = .05), *decision* + 1% (but *p* > .05 on this variable), *discussion* + 5% (*p* = .05), and *writing* AD + 5% (*p* = .03). Moreover, greater age was positively correlated with having written AD at inclusion (21% among retired compared to 2% among young adults) and with the propensity to write AD after our intervention (logistic regression, *p* = .001). Other factors tested (frequency of consultations, gender, level of education) had no effect on participants’ ACP engagement.

**Conclusions:**

When distributed without specific counselling, the tool increased reported ACP engagement, although effect sizes remain modest. Further studies are needed to investigate whether the app could generate greater ACP engagement if used by professionals in dedicated ACP consultations.

**Supplementary Information:**

The online version contains supplementary material available at 10.1186/s12913-023-09593-3.

## Introduction

The continuous development of life-sustaining technologies increases the occurrence of situations where patients and health professionals must choose among different treatment options. However, when a patient has lost her capacity for understanding and has not previously expressed her preferences, health care professionals and surrogates are faced with difficult choices. Advance care planning (ACP) helps to address this difficulty [1, 2]. ACP is defined as a process during which individuals are enabled “to define goals and preferences for future medical treatment and care, to discuss these goals and preferences with family and health care providers, and to record and review these preferences if appropriate” [3]. ACP may involve the writing of advance directives (AD), a document in which patients indicate the treatments and measures to which they consent or do not consent in case of incapacity.

Despite its importance, few patients engage in ACP or write AD [1, 4, 5]. There are various well-documented reasons for this, including that patients often lack knowledge and recognition that ACP is relevant for them, or fail to find appropriate assistance for this psychologically weighty process [5–7]. Even for health care professionals, it can be challenging to initiate and conduct ACP counselling [8, 9]. It is therefore important to find solutions that can accompany citizens and patients throughout the various stages of the ACP process [10, 11]. The stages are as follows: gaining awareness of the issue and acquiring knowledge on the subject; engaging in discussions with relatives and health care professionals; making choices about how they want to be cared for in case of incapacity; informing relevant persons of their choices; finally, documenting those choices in AD.

Barriers to ACP can be addressed in various ways. One promising solution is to exploit easy-to-use and freely available digital technologies such as websites or smartphone applications. These technologies facilitate ACP discussions in family settings, and can be used by professionals during consultations with their patients [12–16]. However, most innovative solutions have only been developed in English [17].

Our interdisciplinary team, composed of ethicists, healthcare professionals, patients-as-partners, and IT experts, developed an app for supporting the ACP process in a Swiss French-speaking context. Details of this work are described in a separate publication [18]. The app is called *Accordons-nous* (*Let’s come to an agreement*). It is primarily intended for patients and relatives, but can also be used by health care professionals as an aid to prepare and support discussions about serious medical events or end-of-life situations.

In line with the key principles of complex intervention development [19], several studies were conducted to obtain feedback on the app from a large population, and to evaluate whether the app was capable of nudging patients to engage in an ACP process. Results of our usability tests were conclusive and are reported in a complementary publication [18]. Here we report efficacy results.

### Study objectives

The primary objective of this study was to conduct an efficacy test of the app *Accordons-nous*. The question was: when this ACP tool is offered to patients in an ambulatory setting, does it motivate them to: a) contemplate ACP issues (e.g. possibility of an accident or worsening health status causing cognitive incapacity); b) make decisions (e.g. about what treatment they would want to receive or not in such situation); c) talk about these decisions with relatives and professionals; d) write their AD? The secondary objective was to test the effect of socio-demographic factors (age, health status, gender, level of education) on patients’ motivation to engage in an ACP process.

## Methods

### Participants

Ambulatory patients varying in age, health status, and socio-economic status were recruited using the criteria described in Table 1.

**Table 1:** Inclusion and exclusion criteria applied to both groups

***Inclusion criteria (on approach):***
- Consultation at a group practice or ambulatory emergency department
- Age ≥ 18 years old
- Estimated time left in waiting room ≥ 30 min
***Exclusion criteria (at first contact):***
- Patient incapable of making decisions about care and therefore unable to write AD
- Patient not showing any interest in the study
- Patient with acute pain at the time of recruitment (to avoid imposing additional burdens on them)
- Patient uncomfortable with using a mobile phone or tablet and without a next of kin who could provide regular help
- Lack of fluency in written and oral French (the app is only available in French)

### Study intervention

Patients were approached individually in medical waiting rooms, the aim and procedures of the study were explained, and written consent was requested. Willing participants were supplied with the consent form and given enough time to read and ask questions before signing. Participants were then instructed to engage with an ACP tool. In the control condition, this was a leaflet containing information about ACP and how to approach the process of writing AD. This leaflet is usually distributed to hospitalized patients at Geneva University Hospitals. In the treatment condition, the ACP tool was the app *Accordons-nous* accessed on an iPad. Participants could consult their ACP tool for 10 min before responding to the first questionnaire. Before leaving, participants were asked to provide contact information and instructed to explore the ACP tool further at home. Control participants were given the leaflet to take away. For treatment participants, the app was installed on their phone, or if time did not permit this, download instructions were provided. 3–4 weeks later, we contacted participants by phone to complete the second questionnaire.

**Fig. 1:**
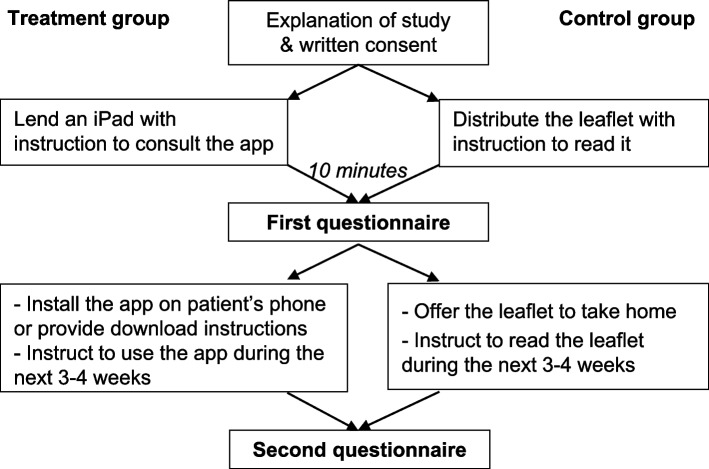
Flow diagram of study procedures

Both questionnaires contained questions related to the study presented here (see details in Table 3 and Additional file 1) and additional questions designed to collect users’ opinions of the usability and content of the tool. The latter data was analyzed in a usability procedure described in a complementary paper, with the aim of improving the design, navigability and content of the tool [18]. The study procedures are described in Fig. 1.

### Setting, recruitment and randomization

Recruitment took place in 6 locations (4 primary care waiting rooms, and 2 ambulatory emergency centres: *University Hospitals of Geneva* and *Clinique et Permanence d'Onex*) from October 2020 to June 2021. 200 to 300 patients were expected to be included during the scheduled time frame.

Seven staff members (2 experienced research nurses and 5 students) recruited participants in both conditions, alternating (within the day or over different days, depending on time at disposal) recruitment sequences with the app (treatment) or the leaflet (control). The decision regarding which condition would be tested was made before going to the site, to avoid any possible influence from the patients present in the waiting room.

Experimenters did not make patient selections during recruitment. All patients meeting the inclusion criteria (≥ 18 years of age, with an estimated waiting time of at least 30 min) were approached in order of arrival. Upon expression of interest, experimenters checked exclusion criteria, and collected signed consent forms before including the patients.

The whole procedure was piloted with two patients-as-partners before actual data collection started. Recruitment staff members participated in training and supervised sessions before recruiting patients on their own once they felt at ease.

### Statistical plan

The dataset includes explanatory variables related to characteristics of participants: *age*, *gender*, level of *education*, number of daily *medications*, frequency of annual *medical consultations*, and type of *recruitment location* (see details in Table 2). Correlation tests (Pearson, Spearman, Kendall, used when appropriate for variable type) were conducted first in order to discard highly correlated variables from subsequent statistical analysis.

**Table 2:** Characteristics of participants. To facilitate comparison of the study population at recruitment and after the intervention, % are calculated within condition (control-leaflet; treatment-application) and study stages (at recruitment; post-intervention)

	At recruitment (*n* = 473)		Post-intervention (*n *= 312)	
	Control	Treatment	Control	Treatment
	*n* = 248	*n* = 225	*n* = 176	*n* = 136
*Age*, mean (median)	45.3 (42.5)	43 (44)	46.4 (45.5)	44.7 (47)
*Gender*, n (%)				
- Female	131 (52.8)	118 (52.4)	94 (53.4)	65 (47.8)
- Male	117 (47.2)	106 (47.1)	82 (46.6)	70 (51.5)
- Other	0	1 (0.4)	0	1 (0.7)
*Education* (highest level achieved), n (%)				
- Elementary school	57 (23)	41 (18.2)	39 (22.2)	27 (19.9)
- High or professional school	93 (37.5)	97 (43.1)	60 (34.1)	53 (39)
- University level	98 (39.5)	87 (38.7)	77 (43.8)	56 (41.2)
*Recruitment location*, n (%)				
- Emergency waiting room	219 (88.3)	205 (91.1)	154 (87.5)	123 (90.4)
- Primary care waiting room	27 (10.9)	19 (8.4)	20 (11.4)	13 (9.6)
- NA	2 (0.8)	1 (0.4)	2 (1.1)	0
*Medication* (nb of daily drugs taken), n (%)				
- 0 drugs	120 (48.3)	109 (48.4)	89 (50.6)	65 (47.8)
- 1–2 drugs	71 (28.6)	78 (34.7)	50 (28.4)	52 (38.2)
- 3–4 drugs	34 (13.7)	27 (12)	19 (10.8)	13 (9.6)
- 5–9 drugs	17 (6.85)	9 (4)	13 (7.4)	6 (4.4)
- 10 and more	6 (2.4)	2 (0.8)	5 (2.8)	0
*Medical consultations* (nb of medical consultations in a year), mean (median)	4.99 (2)	3.97 (2)	4.98 (2)	3.78 (2)

Two-sample tests were used for equality of proportions (Pearson's Chi-square), to test the effect of the app (treatment) compared to the information leaflet (control) on four different dependent ACP-variables: *contemplation* (renewed contemplation after inclusion: yes/no); *decision* (additional decision compared to the baseline at inclusion: yes/no); *discussion* (additional discussion compared to the baseline at inclusion: yes/no); *writing* (additional AD written: yes/no). Logistic regression models were then used to test for the effect of all relevant independent variables (t*reatment*, *age*, *gender*, *medical consultations*, etc.) on the four ACP-variables. The models were iteratively simplified by removing the least significant variables and keeping the model with the lowest Akaike information criterion (AIC) value.

Cluster analysis was not possible with this dataset because the recruitment procedure involved 7 staff members and 6 different locations, generating numerous subsets of unequal sizes. However, a meaningful *recruitment location* variable was created to differentiate participants approached in a primary care waiting room as opposed to an emergency centre waiting room.

A power calculation for the Chi-square test indicated that with a power = 0.90 and sig level = 0.05, an increase of 0.1 points can be observed with *n* = 447, and an increase of 0.2 points with *n* = 263, showing that the intervention was adequately powered to detect small effects with a sample size of more or less 300 participants.

In the preregistration of this study, the intention to check for pre-post and between-group changes on an *ACP sensitivity* scale was announced, calculated as a compound of four ACP subscales (*contemplation*, *decision*, *discussion* and *writing* scales, each ranging between 0 and 1). An internal review procedure led to the decision to use the simpler and more straightforward analyses with binary variables described above. Nevertheless, analyses involving the *ACP sensitivity* scale were also conducted and are presented in the Additional file 1. Results of both types of analyses are highly consistent.

All tests were evaluated for statistical significance at alpha level 0.05. Statistical analysis was performed using R, version 4.1.3.

## Results

### Participant flow

**Fig. 2:**
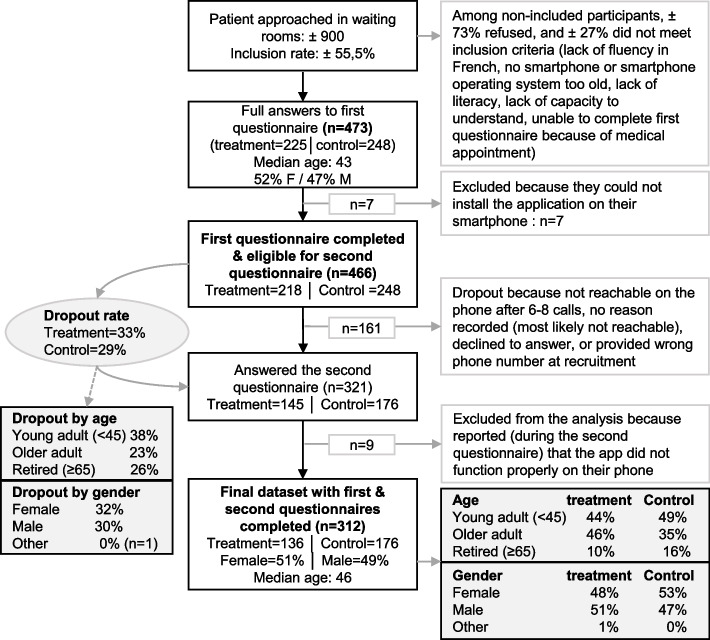
Flow diagram of recruitment and data collection

Due to the COVID pandemic, significant organizational difficulties were encountered at the beginning of the recruitment period. This led to the hire of additional staff. The second wave of recruitment was much more effective than expected because the COVID situation had eased dramatically. More participants were thus recruited than originally planned. In total, approximately 900 patients were approached. This proportion was calculated based on the detailed documentation provided by two recruiters who recruited 67% of the 473 participants. 225 participants were enrolled in the treatment condition and 248 in the control condition. In the course of the study, 6 additional participants asked to be withdrawn from the study. Their data were immediately deleted and are not reported here because there is no record of which stage of the study those withdrawals took place at. The unequal sampling (23 additional participants in the control condition) is due to a miscommunication event at the end of the data collection period. The detailed participant flow is described in Fig. 2.

### Outcomes

#### Written AD at recruitment stage

At recruitment, 5% (*n* = 23/473) of participants reported having written their AD. The same baseline (5% DA at recruitment stage) was observed in the subgroup that completed the study by responding to both questionnaires (*n* = 15/312). The propensity to write AD increases with age, in particular after retirement: young adults < 45y.o., 2% (5/247); older adults > 44, 4% (6/168); retired adults > 64y.o., 21% (12/58).

#### Correlation tests and selection of explanatory variables

The explanatory variables are described in Table 2. The value “primary care waiting room” for the variable *recruitment location* was positively correlated with the number of daily *medications* (*N* = 470, Kendall, *z* = -3.42, *Tau* = -0.15, *p* < 0.001), more *medical consultations* (*N* = 469, Kendall, z = -3.96, Tau = -0.16, *p* < 0.001), and higher *age* (*N* = 470, Kendall,* z* = -4.94, *Tau* = -0.18, *p* < 0.001). In addition, the variable *medication* was positively correlated with *medical consultations* (Spearman, *rs*(470) = 0.39, *p* < 0.001), and *age* (*rs*(471) = 0.35, *p* < 0.001). These two variables were thus discarded in subsequent analyses. No other significant correlations were found. The logistic regressions were thus conducted only with the following set of variables: *treatment*, *gender*, *education*, and *medical consultations*.

#### Interest in the ACP supporting tools (app versus HUG information leaflet)

At the time of the second questionnaire, 55% (*n* = 75/136) participants in the treatment condition reported having consulted the app during the 3–4 weeks after recruitment. In the control condition, 34% (*n* = 60/175) reported having read the information leaflet distributed at recruitment again. This difference in interest in the ACP supporting tool is highly significant (Chi-square test, *DF* = 1, *N* = 311, *X*^2^ = 12.72, *p* < 0.001).

#### Effectiveness of the ACP supporting tools

The effectiveness of the app, compared to the leaflet, on four relevant dependent variables was investigated. The questions asked and main results are described in Table 3.

**Table 3:** Questions asked of participants at the recruitment and post-intervention stages, and main results

**Contemplation** **(of an ACP event)**	*Between group difference (logistic regression): p* = *.048*		
Question asked of all participants:			
*At recruitment:* “Before today, have you ever thought about the possibility of having an accident or a serious illness that would cause you to lose your capacity for judgment? What kind of situation [accident, serious illness] did you think of?”			
*Post-intervention:* “Since the last time we met, have you thought about the possibility of having an accident or a serious illness that would cause you to lose your capacity for judgment? What kind of situation [accident, serious illness] did you think of?”			
**Responses**	**Treatment**	**Control**	**Total Data**
Participants who, at the time of recruitment, reported having contemplated an ACP event	61% (*n* = 83/136)	63% (*n* = 111/176)	62% (*n* = 194/312)
Participants who reported having contemplated an ACP event *during the 3–4 weeks* after inclusion	60% (*n* = 81/136)	49% (*n* = 87/176)	54% (*n* = 168/312)
***Decision* (about contemplated event)**	*Between group difference (logistic regression): p* > *.05*		
Question asked of participants who reported having contemplated an ACP event in the previous question**:**			
*At recruitment & Post-intervention:* “Do you have any idea and can you tell me how you would like to be cared for in such situations?” < referring to the situations described by the patient in the previous question >			
**Responses**	**Treatment**	**Control**	**Total Data**
* At recruitment:* participants who were able to express a decision about how they would like to be treated related to the ACP event(s) considered in the previous question	46% (*n* = 63/136)	40% (*n* = 70/176)	43% (*n* = 133/312)
* Post-intervention:* participants who have expressed (either in the first or in the second questionnaire) a decision about how they would like to be treated related to the ACP event(s)	55% (*n* = 75/136)	48% (*n* = 84/176)	51% (*n* = 159/312)
* Pre-post increase in participants’ expressed* decision about how they would like to be treated related to the ACP event	9% (*n* = 12/136)	8% (*n* = 14/176)	8% (*n* = 26/312)
***Discussion* (about decision)**	*Between group difference (logistic regression): p* = *.047*		
Question asked of participants who reported having taken a decision about an ACP event in the previous question:			
*At recruitment & Post-intervention:* “Have you talked about your life decisions or priorities < referring to the previously discussed event > with someone close to you? And with your professional caregivers?”			
**Responses**	**Treatment**	**Control**	**Total Data**
* At recruitment:* participants who reported having discussed their decision related to the ACP event(s) considered in the previous question with relatives or HCP	35% (*n* = 48/135*)	30% (*n* = 53/175)	32% (*n* = 101/311)
* Post-intervention:* participants who reported having discussed (either in the first or in the second questionnaire) their decision related to the ACP event(s) considered in the previous question with relatives or HCP	49% (*n* = 67/136)	39% (*n* = 68/175)	43%* (n* = 135/312)
* Pre-post increase of participants’ reported discussion* about how they would like to be treated related to the ACP event	14% (*n* = 19/135*)	9% (*n* = 15/175)	11% (*n* = 34/311)
***Writing* (of AD)**	*Between group difference (logistic regression): p* = *.030*		
Question asked of all participants:			
*At recruitment & Post-intervention:* “Have you already written your Advance Directives?”			
**Responses**	**Treatment**	**Control**	**Total Data**
* At recruitment:* participants who reported having written their AD	2% (*n* = 3/136)	7% (*n* = 12/176)	5% (*n* = 15/312)
* Post-intervention:* participants who reported having written their AD	7% (*n* = 9/136)	6% (*n* = 10/176)	6% (*n* = 19/312)
* Pre-post increase in reported written AD*	4% (*n* = 6/136)	-1% (*n* = -2/176)	1% (*n* = 4/312)
* At recruitment:* participants who reported having started to write their AD but not yet finished	0% (*n* = 0/136)	0% (*n* = 0/176)	0% (*n* = 0/312)
* Post-intervention:* participants who reported having started to write their AD but not yet finished	4% (*n* = 6/136)	1% (*n* = 2/176)	2% (*n* = 6/312)

* One missing answer in the first questionnaire

Overall, the intervention (treatment-app and control-leaflet data combined) increased reported ACP engagement on all variables. See details in Table 3.

Baseline *contemplation* rate (participants who reported having already thought about an ACP event): at post-intervention time (3–4 weeks after inclusion), the proportion of participants who reported having (again or for the first time) contemplated an ACP *during the 3–4 last weeks* is higher in the treatment group than in the control group. This between-group difference (+ 11%) is not significant on the 2-sample test for equality of proportion tests (*N* = 312, *DF* = 1, *X*^2^ = 2.77, *p* = 0.096). However, the best fitting multiple logistic regression model (*X*^*2*^ = 21.72, *DF* = 3, *p* =  < 0.001) indicates that, after inclusion, participants receiving the app were significantly more likely to report contemplation compared to those receiving the information leaflet (*OR* = 1.6, 95%CI [1, 2.57], *p* = 0.048). The model also includes the variable *age*, but with a higher p-value (*OR* = 1.01, 95%CI [1, 1.03], *p* = 0.085), providing some indication that, after inclusion, the propensity to report contemplation may increase with age. The full regression model (before iterative removal of least significant variables) produces a similar result: *treatment* (*p* = 0.041) and *age* (*p* = 0.099) are the only variables that are close to significance.

Baseline *decision* rate (participants able to express a decision about how they would like to be treated related to the ACP events considered in the previous question): at post-intervention time, the *decision* rate increased more in the treatment group than in the control group. This between-group difference (+ 1%) is not significant (2-sample test for equality of proportions, *N* = 312, *DF* = 1, *X*^2^ = 0.005,* p* = 0.945). Logistic regression analysis indicates no significant effect of our variables.

Baseline *discussion* rate (participants who reported having discussed their decision related to the ACP event(s) considered in the previous question with relatives or HCP): at post-intervention time, the reported *discussion* rate increased more in the treatment group than in the control group. This between-group difference (+ 5%) is not significant on the 2-sample test for equality of proportions test (*N* = 310, *DF* = 1, *X-squared* = 1.83,* p* = 0.176). However, the best fitting multiple logistic regression model (*X*^*2*^ = 247.36, *DF* = 4, *p* =  < 0.001) indicates that, after inclusion, participants receiving the app were significantly more likely to report ACP discussions compared to those receiving the information leaflet (*OR* = 2.15, 95%CI [1.01, 4.64], *p* = 0.047). The model also includes the variables *age* and *education* but those are not significant. The full regression model containing all variables before simplification produces a similar result: *treatment* is the only significant variable (*p* = 0.041).

Baseline *writing* rate (participants who reported having written their AD): at post-intervention time, the reported *writing* rate increased in the treatment group (4%) but decreased in the control group (-1%). The between-group difference (5%) cannot be directly analyzed with a 2-sample test for equality of proportions because one value of comparison is negative. The two participants who provided contradictory responses (i.e. “I have written AD” in the first questionnaire and “I have not written AD” in the second questionnaire) were thus removed from the analysis. With this adjustment (unfavourable to our test hypothesis), the between-group difference in increase is significant (2-sample test for equality of proportions, *N* = 310, *DF* = 1, *X*^2^ = 5.68,* p* = 0.02), indicating that the app increased participants' motivation to write their AD. In the same line, the best fitting logistic regression model (*X*^*2*^ = 81.7, *DF* = 3, *p* =  < 0.001) indicates a significant increase in reported written AD among participants who received the app as opposed to those that received the information leaflet (*OR* = 1.13, 95%CI [1.79, 2.23], *p* = 0.030). The model also includes the variables *age*, and shows that, after inclusion, the propensity to report written AD was positively affected by increasing age (*OR* = 1.09, 95%CI [1.04,1.15], *p* = 0.001). The full regression model produces a similar result: *treatment* (*p* = 0.021) and *age* (*p* = 0.002) are the only significant variables.

In addition to the increase in reported written AD (compared to the baseline at recruitment), an increase in participants who reported having started to write their AD but not yet completed the process is observed. Again, this increase was larger in the treatment group (4%) than in the control group (1%).

## Discussion

Overall, providing an ACP tool (app or leaflet) to participants in a medical waiting room increased ACP engagement. The app had a larger positive effect compared to the leaflet usually provided at the Geneva Hospitals. The difference is significant regarding participants’ rate of reported contemplation of an ACP event, discussion about their treatment preference in case of that ACP event, and writing AD. In particular, a 4% increase in reported written AD in the app group and no increase in the leaflet group is observed. Moreover, an additional 4% of participants in the app group reported having started to write their directives compared to 1% in the leaflet group. This positive effect is probably due to the fact that the app contained informative and captivating content and an easy-to-understand AD form that could be directly filled in. The information leaflet contained more formal information and only a link to download an AD form from the Internet. It is well-known that readable and discussion-prompting content, as well as accessibility, are effective ways to promote the completion of AD [14]. These results provide further confirmation of this.

The results of the study confirm that age, which is correlated with the number of daily medications taken, is a motivating factor for engaging in ACP. Few patients had written their AD at recruitment stage (5%) with a higher proportion among the subgroup of retired patients (21%). This is consistent with other studies [20]. Moreover, participants with greater age reported significantly more writing of AD after the intervention. This result is a confirmation that the technological aspect of the app is not a disincentive for elderly participants with greater age.

Participants’ feedback on the tool collected in this study was used to refine the development of the app and assess its usability. These data, added to further usability results, are reported in a separate paper [18] and provide positive results regarding the tool’s navigability, comprehensiveness, and perceived relevance. At a quantitative level, the fact that 21% more participants reported having consulted the app than the leaflet is a further sign of interest in this ACP tool.

None of the other factors included in the analyses (*gender*, level of *education*, frequency of *medical consultations*) affected the motivation of our participants to engage in ACP when the app or leaflet was presented to them. These results may indicate that efforts to raise awareness towards ACP are likely to have a similar impact on men or women, or on patients with different education or health statuses. However, answering this was not the aim of the study. These factors were included in study models mainly to detect possible confounding variables.

In some cases, inconsistent responses between the first and the second questionnaire were obtained. Notably, a few participants in the control condition reported at recruitment that they had written AD, but 3–4 weeks later, responded they had not written AD. After analyzing these cases, there are three possible explanations. Participants may have been confused about what AD meant at the recruitment stage despite experimenters providing relevant explanations. For instance, they may have talked about ACP issues with family or health professionals and wrongly believed that this counted as having completed an AD. Later, they may have realized that AD is a formal document and thus acknowledged not having written AD in the second questionnaire. Alternatively, participants may have responded negatively at the second stage because they thought that we asked them whether they had updated their AD during the limited 3–4 weeks’ time frame between recruitment and the second questionnaire. Alternatively, inconsistent responses may be the effect of a courtesy bias: the willingness to answer positively in order to please the experimenter may have been stronger during the first questionnaire conducted in their presence than during the second questionnaire when participants answered on the phone.

Overall, the effect sizes reported here are small, especially those on self-reported ACP decisions, which are not statistically significant. Before conducting the study, larger effects were expected with the use of the app, especially on patients with a weaker health status (e.g. those with greater age or needing more medication and medical consultations). ACP tools may need to be accompanied by active medical counselling provided by professionals in order to exert stronger effects. This has been demonstrated in further studies exploring factors that facilitate the ACP process with patients [21]. The most successful interventions incorporated direct patient–health professional interactions over multiple consultations. However, ACP counselling could be facilitated and its quality enhanced if supported by the use of an app such as *Accordons-nous*. Indeed, many studies have shown that it is difficult for health care professionals to start the discussion process, and that they lack the resources of support and time [22–26]. The app could serve as an icebreaker, as a catalyst for ACP discussions, and as a documentation facilitator, accompanying a multi-step process involving patients, family and health care providers.

### Limitations

By design, the *decision* question (“Do you know how you would like to be cared for in such situations?”) was asked only upon a positive answer to the *contemplation* question (‘Yes’ to “Have you thought about the possibility of having an accident or a serious illness that would cause you to lose your capacity for judgment?”). Following the same logic, the *discussion* question (“Have you talked about your decisions or priorities with…?”) was asked only upon a positive answer to the *decision* question. Thus, some of the participants may not have had the opportunity to report all their ACP behaviour. For instance, if they had discussed their life priorities with relatives or health care professionals without any particular ACP events in mind, the design of the questionnaire did not allow them to report it. In the statistical analysis, “Question not asked” was coded as a “No” response. This methodological choice should be kept in mind while interpreting the results. The results may underestimate more broadly conceived decision and discussion behaviour.

The app installed on participants’ phones was a nearly-finished test version. The tester mode involved some technical difficulties (partly unforeseen) related to the installation of the app, specifically on Android devices. This technical difficulty is now resolved but at the time of data collection, it caused a series of dropouts in the treatment condition.

Patients were mainly recruited in emergency centre waiting rooms. Participants’ quality of attention while responding to the first questionnaire may have been affected by their personal medical condition.

## Conclusions

The research shows the efficacy of an app for promoting engagement in ACP among patients, including patients with greater age. In this study, the app was distributed without specific counselling in an ambulatory medical setting. The next step would be to evaluate the efficacy of the tool when used in the context of a structured patient management procedure. Patients with greater age or suffering from severe illness may benefit the most from ACP consultations held with this supporting tool. Follow-up work could evaluate whether health care professionals find the app appropriate and useful for initiating and conducting ACP counselling with their patients.

### Supplementary Information

**Additional file 1.** Related to the paper “Effect of an App for Promoting Advance Care Planning and Motivating Patients to Write their Advance Directives”.

## Data Availability

The study was preregistered on the OSF platform (https://osf.io/s9zjw) on 22.04.2021. The dataset generated and analyzed during the current study and the material and codes are available on the OFS repository, https://osf.io/r3hmp/?view_only=341d6ced7fc74a2ea24ab94f1816f9f8.
