# Supplementary material for: Effect of an app for promoting advance care planning and motivating patients to write their advance directives
Source: BMC Health Serv Res. 2023 Jun 1;23:566. doi: 10.1186/s12913-023-09593-3 (PMC10233869; doi:10.1186/s12913-023-09593-3)
Supplement: Supplementary file 1 — Additional file 1. Related to the paper “Effect of an App for Promoting Advance Care Planning and Motivating Patients to Write their Advance Directives”. [file 12913_2023_9593_MOESM1_ESM.docx]

**Additional File 1**

Related to the paper “Effect of an App for Promoting Advance Care Planning and Motivating Patients to Write their Advance Directives”

Content

[Additional statistics 1](#_Toc135298374)

[Questionnaire in English (translation) 5](#_Toc135298375)

### Additional statistics

In the preregistration of this study (https://osf.io/s9zjw), the intention to check for pre-post and between-group changes on an *ACP sensitivity* scale, calculated as a compound of four ACP subscales (*contemplation*, *decision*, *discussion*, *writing*) was announced. Here is the result of these analyses.

**Method**

In these additional analyses, the variables were computed as ordinal scales, by taking more collected information into account and making more assumptions (described in Supplementary Table 1). Four ACP sub-subscales were created, each ranging between 0 and 1: *contemplation* scale, *decision* scale, *discussion* scale, and *writing* scale. The general *ACP sensitivity* scale, ranging between 0 and 4, was a compound of the four subscales.

| **Supplementary Table 1. Coding logic used to compute the four subscales.** | | | |
| --- | --- | --- | --- |
| **Variable** | **Range of scores** | **Based on answers to the following question(s)** | **Coding logic** |
| ***Contemplation* scale** | 0-1 | [Question asked of all participants]  **At recruitment**  “Before today, have you ever thought about the possibility of having an accident or a serious illness that would cause you to lose your capacity for judgment? What kind of situation did you think of?”  **Post-intervention**  “Since the last time we met, have you thought about the possibility of having an accident or a serious illness that would cause you to lose your capacity for judgment? What kind of situation did you think of?” | **At recruitment**  No = 0  Yes = 0.5  **Post-intervention**  In addition to pre-intervention score:  Yes = + 0.5  No = + 0 |
| ***Decision* scale** | 0-1 | [Question asked of participants who reported having contemplated an ACP event in the previous question]  **At recruitment & Post-intervention:**  “Do you have any idea and can you tell me how you would like to be cared for in such situations?” <referring to the situations described by the participant in the previous question> | **At recruitment**  Response = “No” or question not asked = 0  Response = “Yes” but vague explanation unlikely to be of use to HCP (ex: “I do not want overtreatment”, “it depends on the situation”, “I guess the caregivers will make the decision”) = 0.5  Response = “Yes” + the explanation provided is relevant for HCP = 1  **Post-intervention**  If question not asked, report pre-intervention score  If new answer, use same coding logic as in pre-intervention |
| ***Discussion* scale** | 0-1 | [Question asked of participants who reported having taken a decision about an ACP event in the previous question]  **At recruitment & Post-intervention:**  “Have you talked about your life decisions or priorities < referring to the previously discussed event > with someone close to you? And with your professional caregivers?” | **At recruitment**  Response = “No” or question not asked = 0  Response = “Yes with a relative” = +0.25  Response = “Yes with a health care professional” = +0.25  **Post-intervention**  In addition to score obtained at recruitment:  Response = “No” or question not asked = +0  Response = “Yes, an additional discussion with a relative in the 3-4 last weeks” = +0.25  Response = “Yes, an additional discussion with a health care professional in the 3-4 last weeks” = +0.25 |
| ***Writing* scale** | 0-1 | [Question asked of all participants]  **At recruitment & Post-intervention:**  “Have you already written your Advance Directives?” | **At recruitment**  Response = “No” or “I did it orally” = 0  Response = “Not finished but in writing process” = 0.5  Response = “Yes” = 1  **Post-intervention**  Same coding logic as at recruitment |
| ***ACP sensitivity* scale** | 0-4 |  | Sum of the four subscales |

The overall effect of the intervention (difference in score between recruitment and post-intervention stages) was checked on the global *ACP sensitivity* scale, and on the four ACP subscales separately with paired Student's t tests. These tests were performed on the whole dataset (treatment and control together) and on the control and treatment dataset separately.

The variables *medication* and *recruitment location* were discarded based on the correlation tests described in the main paper, and further correlation checks between all the remaining explanatory variables (*treatment*, *age*, *gender*, *medical consultations*, *education*) were undertaken.

The independent effects of the explanatory variables were tested with linear mixed models, taking in turn the *ACP sensitivity* scale, and the four ACP subscales as dependent variables. The best fitting models were selected by iteratively removing nonsignificant explanatory variables and keeping models with higher adjusted R^2^ and lower residual standard error.

All tests were evaluated for statistical significance at alpha level 0.05. Statistical analysis was performed using R, version 4.1.3.

**Results**

No significant correlation was found between the explanatory variables (*treatment*, *age*, *gender*, *medical consultations*, *education*).

Overall *ACP sensitivity* scores increased significantly after the intervention, overall (*M_t0_*=.82, *M_t1_*=1.25, paired t-test: *t*(309)=12.55, p<.001), as well as in the control condition (*M_t0_*=83., *M_t1_*=1.18, paired t-test:*t*(174)=8.39, *p*<.001) and in the treatment conditions (*M_t0_*=80., *M_t1_*=1.36, paired t-test:*t*(134)=9.54, *p*<.001). A larger mean increase in the treatment (.55) than in the control (.35) condition is thus observed. Results of the best fitting multiple linear regression model indicate a collective significant positive effect of *Treatment*, and *age* (*F*(3/306)=175.9, *p*<., *R^2^*=.63, *R^2^_adj_*=.63) on *ACP sensitivity* scores: more specifically, receiving the app as opposed to the information sheet (*t*=3.01, *SE*=07, *p*=003) and increasing age (*t*=2.38, *SE*=002., *p*=018) are significant predictors.

*Contemplation* subscores increased significantly after the intervention, overall (*M*_t0_=.31, *M*_t1_=.58, paired t-test: *t*(311)=19,05, *p*<.0001), as well as in the control condition (*M*_t0_=.32, *M*_t1_=.56, paired t-test:*t*(135)=14.1, *p*<.001) and in the treatment conditions (*M*_t0_=.31, *M*_t1_=.60, paired t-test:*t*(175)=13.08, *p*<.001). A larger mean increase in the treatment (.30) than in the control (.25) condition is thus observed. Results of the best fitting multiple linear regression model indicate a collective significant positive effect of *treatment*, and *age* (*F*(3/308)= 158.8, *p*<.001, *R^2^*=.61, *R^2^_adj_*=.60) on *contemplation* subscores: more specifically, receiving the app as opposed to the information sheet (*t*=1.99, *SE*=.03, *p*=.047) and increasing age (*t*= 1.73, *SE*<.001, *p*=.085) are significant predictors.

*Decision* subscores increased significantly after the intervention, overall (*M_t0_*=.37, *M_t1_*=.44, paired t-test: *t*(311)=3.55, *p*< .001), as well as in the control condition *(M_t0_*=.36, *M_t1_*=.42, paired t-test:*t*(175)= 2.01, *p*<.046) and in the treatment conditions (*M_t0_*=.38, *M_t1_*=.48, paired t-test:*t*(135)=3.05, *p*<.002). A larger mean increase in the treatment (.10) than in the control (.05) condition is thus observed. This difference, however, does not come out as significant (*t*=1.34, *SE*=.04, *p*=.182) in the best fitting linear regression model (*F*(2/309)=134.9, *p*<.001,*R^2^*=.47, *R^2^_adj_*=.46).

*Discussion* subscores increased significantly after the intervention, overall (*M_t0_*=.09, *M_t1_*=.16, paired t-test: *t*(309)=9.89, *p*<.001), as well as in the control condition (*M_t0_*=.09, *M_t1_*=.15, paired t-test:*t*(174)=6.52, *p*<.001) and in the treatment conditions (*M_t0_*=.09, *M_t1_*=.18, paired t-test:*t*(134)=7.53, *p*<.001). A larger mean increase in the treatment (.09) than in the control (.05) condition is thus observed. Results of the best fitting multiple linear regression model (*F*(6/303)=142.9, *p*<.001, *R^2^*=.74, *R^2^_adj_*=.73) indicate that the *treatment* condition (*t*=2.53, *SE*=.01, *p*=.012) is a significant predictor of *discussion*.

*Writing* subscores increased significantly after the intervention, overall (*M_t0_*=05., *M_t1_*=.07, paired t-test: *t*(311)=2.43, *p*<.016), as well as in the control condition and in the treatment conditions (*M_t0_*=.02, *M_t1_*=.09, paired t-test:*t*(135)=3.4, *p*<.001), but not in the control condition (*M_t0_*=.07, *M_t1_*=.06, paired t-test:*t*(175)=-.53, *p*=.59). No relevant effect in the control condition and a large mean increase in *writing* scores in the treatment condition (.07) is thus observed. Results of the best fitting multiple linear regression model indicate a collective significant positive effect of *treatment* and *age* (*F*(6/304)=54.36, *p*<.001, *R^2^*=.52, *R^2^_adj_*=.51) on *writing* subscores: more specifically, receiving the app as opposed to the information sheet (*t*=3.29, *SE*=.02, *p*=.001) and increasing age (*t*=3.82, *SE*=.001, *p*<.001) are significant predictors.

Except for the effect reported above, no further effects were found. Notably, *gender,* level of *education*, and frequency of *medical consultations* did not affect any of the ACP engagement scores in the dataset.

The results produced in this supplementary analysis are fully coherent with those presented in the main article.

| Questionnaire in English (translation) | | | | |
| --- | --- | --- | --- | --- |
| **At recruitment** | | | | |
| Recruitment location: [HUG ; CPO ; Vesenaz, SaintJean, Lancy, Autre] | | | | |
| Date of signature of the consent form : [Year, month, day] | | | | |
| Investigator: [JB ; DS ; CB ; CS ; SC, AlP, AP] | | | |  |
| Which group is it? [Treatment-Application; Control-leaflet] | | | | |
| How do you define your gender? [M=Male; F=Female; O=Other] | | | | |
| How old are you? [num]  What is your educational background (the main qualification(s) you have obtained)? [Elementary school, High school, Apprenticeship, Business school, University/Higher education, Other[text]] | | | | |
| In the last few weeks, how many (type of) drugs have you taken per day? [None; 1 to 2; 3 to 4; 5 to 9; 10 or more] | | | | |
| In the last 12 months, how many times have you consulted a doctor (GP, specialist, etc.)? [num] | | | | |
| Before today, have you ever thought about the possibility of having an accident or a serious illness that would cause you to lose your capacity for judgment? What kind of situation [accident, serious illness] did you think of? [YES; NO] | | | | |
|  | If YES, |  |  |  |
|  | What kind of situation did you think of? [list text boxes + instruction of investigator: *for each item, tick the label 'accident' or 'illness'*] | | | |
|  | Do you have any idea (however vague) of how you would like to be treated medically in such situations? [YES; NO] | | | |
|  | If YES, |  |  |  |
|  | Can you tell me how you would like to be treated in such situations? [text] | | | |
|  | Have you talked about your life decisions or priorities < referring to the previously discussed event > with someone close to you? [YES; NO] | | | |
|  |  | If YES, what kind of relative? [Spouse or life partner, child, parent, friend, sibling, Other [text]] | | |
|  | Have you talked about your life decisions or priorities with your professional caregivers? | | | |
|  |  | If YES, what kind of caregiver? [GP, home nurse, therapist, hospital doctor, hospital nurse, other [text]] | | |
| Have you already written your Advance Directives? [YES; Yes-but not finished; No-not written; No-but I did it orally; No-never heard of AD] | | | | |
| Can you give us your contact details so that we can get back to you in 3-4 weeks (e-mail and/or telephone number)? [text], [num] | | | | |
| Which time slots (days, hours) would be most convenient for our telephone interview? [text] | | | | |
|  | | | | |
| **Post-intervention questions** | | | | |
| Number of call attempts: [num] | | |  |  |
| Which group is it? [Treatment-Application; Control-leaflet] | | | | |
| Since the last time we met, have you thought about the possibility of having an accident or a serious illness that would cause you to lose your capacity for judgment? What kind of situation [accident, serious illness] did you think of? [YES; NO] | | | | |
|  | If YES, |  |  |  |
|  | What kind of situation did you think of? [list text boxes + instruction of investigator: *for each item, tick the label 'accident' or 'illness'*] | | | |
|  | Do you have any idea (however vague) of how you would like to be treated medically in such situations? [YES; NO] | | | |
|  | If YES, |  |  |  |
|  | Can you tell me how you would like to be treated in such situations? [text] | | | |
|  | If YES, |  |  |  |
|  | In the last 3-4 weeks, have you talked about your life decisions or priorities < referring to the previously discussed event > with someone close to you? | | | |
|  |  | If YES, what kind of relative? [Spouse or life partner, child, parent, friend, sibling, Other [text]] | | |
|  | In the last 3-4 weeks, have you talked about your life decisions or priorities < referring to the previously discussed event > with someone close to you? | | | |
|  |  | If YES, what kind of caregiver? [GP, home nurse, therapist, hospital doctor, hospital nurse, other [text]] | | |
| Have you written your Advance Directives or updated them? [YES; Yes-but not finished; No-not written; No-but I did it orally; No-never heard of AD; Updated] | | | | |
| Have you reused/read the app/booklet again? [YES; NO] | | | |  |
|  | | | |  |
